# Traumatic Brain Injury, Sleep Disorders, and Psychiatric Disorders: An Underrecognized Relationship

**DOI:** 10.3390/medsci6010015

**Published:** 2018-02-15

**Authors:** Anne M. Morse, David R. Garner

**Affiliations:** 1 Janet Weis Children’s Hospital, Department of Pediatric Neurology and Sleep Medicine, Geisinger Medical Center, MC 14-12, 100 N Academy Blvd, Danville, PA 17822, USA; 2 Department of Pediatrics, Geisinger Medical Center, Danville, PA 17822, USA; Drgarner@geisinger.edu

**Keywords:** traumatic brain injury, anxiety, depression, post-traumatic stress, attention deficit disorder, sleep–wake disorders

## Abstract

Traumatic brain injury (TBI) is commonplace among pediatric patients and has a complex, but intimate relationship with psychiatric disease and disordered sleep. Understanding the factors that influence the risk for the development of TBI in pediatrics is a critical component of beginning to address the consequences of TBI. Features that may increase risk for experiencing TBI sometimes overlap with factors that influence the development of post-concussive syndrome (PCS) and recovery course. Post-concussive syndrome includes physical, psychological, cognitive and sleep–wake dysfunction. The comorbid presence of sleep–wake dysfunction and psychiatric symptoms can lead to a more protracted recovery and deleterious outcomes. Therefore, a multidisciplinary evaluation following TBI is necessary. Treatment is generally symptom specific and mainly based on adult studies. Further research is necessary to enhance diagnostic and therapeutic approaches, as well as improve the understanding of contributing pathophysiology for the shared development of psychiatric disease and sleep–wake dysfunction following TBI.

## 1. Introduction

Traumatic brain injuries (TBI) are common in the pediatric population and can have neurocognitive consequences. Understanding the factors that influence risk for a child or adolescent to experience a TBI is an important first step in exploring the consequences of TBI. Several studies have reported specific risk factors, including pre-existing psychiatric and behavioral problems to increase the likelihood to sustain a traumatic brain injury in the pediatric population (Table 1). For instance, recent studies have shown that attention deficit hyperactivity disorder (ADHD), aggression, psychiatric prescription medication use, and use of mental health services increase the risk of TBI [1,2]. These factors have been ascertained by both prospective and retrospective analysis. The results of these findings highlight some overlap, but also identify some discrepancy in risk factors, leading one to question the influence of recall bias, influence of etiology of TBI or other contributing factors to these differences (Table 2) [1,2,3,4,5,6,7]. 

Post-concussive syndrome (PCS) is defined by symptoms occurring after a head injury including, but not limited to, somatic, sleep, cognitive and/or emotional/behavioral difficulties (Table 3) [10,11,12,13,14]. It was previously thought that approximately 15% of those who suffer a single mild TBI (mTBI) will develop chronic PCS; however, McInnes found that this number is likely significantly higher [15]. In fact, a large proportion will continue to have a measurable impairment more than a year out from the injury [15]. In 2012, an estimated 329,290 children, younger than 20, were treated in United States emergency departments for TBI [16]. Among this demographic, the rate of emergency department visits for sports and recreation-related injuries with TBI more than doubled between 2001 and 2012 [16]. In fact, approximately 20% of 8–12th grade students were identified as having had at least one concussion, with 5.5% sustaining recurrent injuries [3]. It is important to identify associated risk factors for TBI within this group, as well as contributors to successful recovery to improve incidence and reduce morbidity.

The clinical course following TBI is influenced by multiple factors. Pre-injury behavior and functioning are strong predictors for the long-term development of behavioral problems and worsening of symptoms of psychiatric disorders [24,25,26]. For instance, children experiencing a significant life stressor prior to injury have been found to be at greater risk of persistent post-concussive symptoms after TBI [13,27]. Children with behavioral problems are commonly endorsed as being at greater risk for experiencing a TBI; however, these specific behavioral disorders are not commonly well defined [4,13]. 

**Figure 1:**
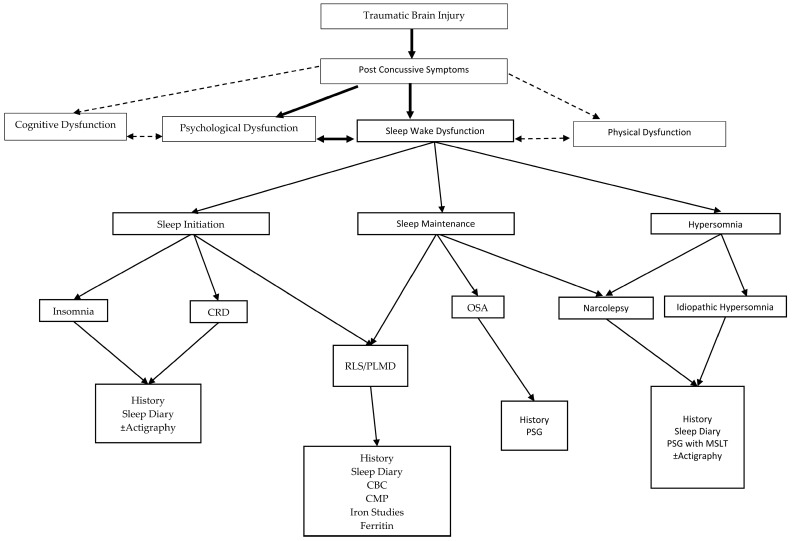
Traumatic brain injury and the development of post-concussive syndrome, highlighting the development of sleep wake dysfunction and its relationship to co-morbid PCS symptoms. CRD: Circadian Rhythm Disorder; CBC: Complete Blood Count; CMP: Complete Metabolic Panel; OSA: Obstructive Sleep Apnea; PSG: Polysomnography; MSLT: Multiple Sleep Latency Test; RLS: Restless Leg Syndrome; PLMD: Periodic Limb Movement Disorder.

In addition to baseline behavioral and psychiatric features, sleep–wake dysfunction is also associated with TBI (Figure 1). Sleep difficulties can affect cognition (particularly attention, memory, and executive functions), behavior, and emotional problems (Table 4) [28]. Pre-existing sleep conditions enhance the likelihood of experiencing post-concussive symptoms [29]. In addition, the presence of a comorbid sleep disorder contributes to psychologic instability, resulting in increased emotional lability and behavioral problems with worsened daily executive function [28,30]. In general, symptoms from a mild TBI should disappear by 3 months, and functional status improves over the first six to twelve months without obvious regression over the first 30 months [31]. However, in patients with history of psychiatric disease and/or sleep dysfunction, recovery may be more protracted (Table 5) [10]. 

The complexity in care of TBI patients is reflective of the multi-disciplinary needs of these patients. TBI-related morbidity may be improved with enhanced understanding of factors that not only contribute to risk for experiencing TBI, but also improved understanding and assessment of the post-concussive factors that influence the course of recovery. This manuscript will focus on the relationship of sleep and psychiatric features. It will explore the clinical relationship, examine the possible crossover in pathophysiology of TBI, sleep, and psychiatric disorders, and discuss the approaches to consider for diagnosis and treatment, highlighting the need for a comprehensive multidisciplinary evaluation to improve recovery times and outcomes [44]. 

## 2. Anxiety

Children with TBI are at a significantly higher risk than those with orthopedic injuries to present with new-onset mood and/or anxiety disorders [45]. Anxiety can be defined as the brain’s response to danger causing avoidance type behavior [46]. Age at time of injury may influence the development of symptoms. Children who are younger than 10 years old are at higher risk of developing post-concussive anxiety disorders [23,47]. There is also a relation to sleep–wake dysfunction. 

In general, youth with anxiety are found to have an increased rate of sleep problems with 88% of those with anxiety reporting at least one sleep problem, and 55% reporting three or more sleep problems [48]. One study also showed that sleep disturbance may vary with age, with younger children being more prone to nighttime wakings and sleep anxiety, and adolescents being more likely to experience excessive daytime sleepiness [49]. After TBI, individuals with continued symptoms of insomnia and fatigue, up to 2 years after the injury, have been found to have higher rates of depression and anxiety [50]. In fact, sleep disturbance, even in the acute post-TBI period, predicted the development of anxiety and depression in the chronic period for all severities of TBI. 

## 3. Major Depressive Disorder

Depressive disorders occur in 10–25% of children post TBI [39,51,52]. Children greater than 12 years of age are five times more likely to experience post-traumatic depressive symptoms [51]. In general, depressed patients may have problematic sleep, as well cognitive difficulties and energy loss. Insomnia or hypersomnia is frequently one of the defining characteristics for depression. The development of depressive symptoms has been suggested to be due to either the injury itself, or as a result of other post-concussive comorbidities, such as anxiety, aggression, and sleep disturbance [53,54]. 

Due to their intimate relationship, the symptoms of depression and sleep–wake dysfunction influence the development and prognosis of one another. Patients identified to have a sleep disturbance ten days post TBI were 6 times more likely to have depression [54]. Sleep deprivation, defined as 6 h of sleep or less a night, in adolescents at baseline, had a 25–38% increased risk of developing depressive symptoms at follow up exams [55]. On the other hand, major depression and depressive symptoms increase the risk for the development of insomnia [56]. There is a suggestion that early onset depression may be related to the direct injury, whereas late onset depression may be due to a psychological reaction to the injury [57]. However, when including sleep symptoms as part of the evaluation, the pattern of development is less clear.

## 4. ADHD

ADHD has not only been shown to increase the likelihood to experience TBI, but a preexisting diagnosis of ADHD may also lead to worse outcomes after TBI [58,59,60]. ADHD that develops as a result of head injury is referred to as secondary ADHD or S-ADHD [61]. S-ADHD has been shown to develop in about 10–20% of patients post TBI. In one study, 15% of S-ADHD cases developed after one year and 21% after two years [9]. Increased TBI severity also increases the incidence of S-ADHD from 7–46% going from mild to severe TBI respectively [51]. Children with TBI, less than 2 years old, have double the risk for the development of S-ADHD as compared to the general population [62], thus raising the questing as to whether S-ADHD is a direct result of injury versus a biased population of children with poor self-regulation who may be more likely to participate in risk-taking behaviors resulting in injury [62]. 

The relationship between sleep and ADHD has a significant bidirectional effect, which is exaggerated in children following TBI [63]. Children with TBI and ADHD have a poorer sleep quality and quality of life than children with primary ADHD without TBI [64]. Evaluation of comorbid sleep dysfunction in secondary ADHD is lacking, but likely has a similar deleterious effect. 

## 5. Post-Traumatic Stress Syndrome

Post-traumatic stress disorder (PTSD), as a part of PCS, has been well described in adults with TBI [65]. Data is lacking, however, to demonstrate the same relationship in children. Specific symptoms of PTSD present after TBI have been poorly defined in pediatric studies. There is a suggestion that children with orthopedic injuries more frequently display PTS symptoms than those with mTBI and met more symptom criteria at 12 months [40]. On the other hand, there is also a report of children with severe TBI exhibiting higher levels of PCS symptoms than those with moderate TBI or orthopedic injury [66]. Furthermore, another study demonstrated that childhood PTSD after traffic injuries was associated more with increasing age and parental PTSD, and no relationship to severity of injury [67]. The evolving anatomy and age-specific biomechanical properties of the developing child increase risk for distinct types of injuries that rarely occur in adults [68]. This may contribute to the differences observed in the development of PTS symptoms between adults and children. 

Another ill-defined parameter in the literature is what symptoms of PTSD are present post TBI [69]. Persistent attention deficits at 3 months post injury have been identified as a risk factor for continued PTS symptoms at 6 months [70]. On the other hand, working memory and verbal learning deficits were found to be protective [70]. This may suggest that patients with impaired working memory and verbal learning, have impaired ability to recall the event, lending to reduced PTS symptoms [65]. Many comorbid conditions often present with TBI and can lead to shorter life expectancy, poor academic performance, and neurocognitive deficits [71]. It has been suggested in adults that sleeping difficulties may be an earlier indicator for risk of PTS disorder [72]. Another study with veterans notes that nightmares are commonly comorbid with TBI [73]. Those with insomnia and PTSD post TBI were found to have a subjective increase in sleepiness as compared to those with just PTSD and insomnia [73]. Literature evaluating sleep-specific risks associated with PTSD in pediatrics is lacking for comparison. 

## 6. Crossover Pathophysiology of TBI, Sleep, and Psychiatric Disorders

TBI can be the result of diffuse or focal injury and frequently can be a combination of both. Diffuse injury occurs when the mechanism causes non-specific global damage, as in diffuse axonal injury or concussion. Focal injury occurs when the mechanism causes a specific targeted area of damage, such as with hematoma or contusion. These injuries may be a result of direct linear force (coup), acceleration deceleration forces (contra-coup) or a combination of both, causing shearing injuries and axonal damage [68]. In addition, there are secondary brain injuries that develop over hours to days that may result from perfusion abnormalities, neuroinflammation, excitotoxicity and dysregulated cell signaling [74,75]. 

The frontal–striatal circuits, which can affect executive function and wakefulness are particularly vulnerable [76]. Damage to this system is found in 18–38% of children who have suffered a TBI between the ages of 5–15 and may be related to the impaired executive function identified in the first year after injury [77]. Emotional dysregulation can also be common after TBI; this combined with executive dysfunction and hormonal imbalance can make adolescents who experience TBI more susceptible to impulsive decisions and poor choices in social situations [78]. This also may indicate why performance on neuropsychological testing may be normal; however, patients still experience significant functional impairment in real-world situations [78]. 

Sleep–wake dysfunction following TBI is common, affecting up to 70% of patients. The sleep–wake cycle is tightly controlled via cooperation between circadian rhythms, sleep–wake homeostasis, and external environmental factors such as medication, diet, stress, and surroundings [37]. The main sleep-promoting pathways are found in the ventrolateral (VLPO) and median preoptic nuclei (MnPO), which inhibit ascending arousal pathways in the brainstem and hypothalamus [79]. The arousal areas include histaminergic tuberomammillary nucleus, orexinergic lateral hypothalamus, noradrenergic locus coeruleus, serotonergic dorsal raphe, and the cholinergic laterodorsal tegmental and pedunculopontine tegmental nuclei [80]. 

Post-mortem evaluations of the brains of patients with and without TBI demonstrated a significant reduction in hypocretin neurons [81,82]. Impaired hypocretin (orexin) signaling causes excessive daytime sleepiness [37,83,84]. It has been shown that reduced cerebrospinal fluid (CSF) orexin levels are associated with a worse clinical outcome with greater likelihood for depression and sleep–wake dysfunction [85,86]. 

Impaired melatonin production has also been suggested to be contributory [81]. Melatonin directs this circadian regulation of sleep and wakefulness, but also has been found to have anti-inflammatory properties [37]. Melatonin may repress TBI-induced inflammation by activating mitophagy and removing damaged mitochondria [87], although it secretes directly into the third ventricle and levels can be much higher in the CSF than in the peripheral blood [88]. Peripheral sampling does provide an accurate surrogate. Melatonin production can be impacted by TBI. CSF melatonin may vary depending on time from TBI. Acutely, there is evidence of increased melatonin with decreased levels as time progresses [81,89]. These findings, however, have been inconsistent. The acute increase is suggested to be related to the anti-inflammatory properties, which may contribute to neural recovery [89,90]. Additionally, this variation may be related to the spectrum of post-traumatic sleep disorders seen (i.e., hypersomnolence to delayed sleep phase disorder) [81,91].

Circadian rhythm is associated with mood regulation, and disturbances can be linked to the development of psychiatric symptoms [37,92]. There has been the suggestion that this may be related to clock genes, which regulate circadian entrainment. Certain clock genes have been implicated in altering the homeostasis of individuals leading to psychiatric disorders such as autism, ADHD, anxiety, major depressive disorder, bipolar disorder, and schizophrenia [93]. This may represent an increased genetic susceptibility for the development of comorbid post-traumatic sleep dysfunction and mental illness. 

## 7. Evaluation and Treatment Options

A multi-disciplinary approach should be taken in the clinical evaluation of patients following TBI. The consideration of specialties to be involved include neurology, psychiatry, sleep medicine, rehab services, social work and sports medicine, depending on the mechanism of injury. There should be a standardized intake, such as the acute concussion evaluation [10], to ensure a comprehensive evaluation of symptoms. Establishing a pre-morbid baseline may be helpful in stratifying risk for the development of PCS. In addition, it is important to identify patient-perceived impact of head injury and goals for recovery. 

Treatment of sleep or psychiatric disorders post TBI is mainly based on adult studies, with limited information on treatment in pediatrics. Frequently, the treatment applied is based on recommendations that have been successful in the relevant psychiatric and sleep disorder in the non-traumatic brain injury population [94]. The approach to treatment in patients with comorbid sleep and psychiatric dysfunction should address symptoms of both processes.

In general, psychiatric medications in pediatrics are started with the lowest dosing and titrated slowly, as pediatric patients may be more susceptible to side effects of these medications [94,95,96,97]. The selection of medication is based on the psychiatric symptoms present (Table 6). Selective serotonin reuptake inhibitors are considered first-line treatment for anxiety and depression [94]. S-ADHD treatment with stimulant medication has been shown to likely be beneficial; however, there seems to be a more attenuated response for S-ADHD than that of primary ADHD [2,98,99]. Of note, those treated with psychostimulant medication prior to TBI, have been noted to have a lower risk of TBI [100]. In fact, retrospectively it was identified that most ADHD patients who sustained TBI were not pharmacologically treated prior to the injury [61,101]. 

PTS disorder patients with nightmares have been shown to have improvements with prazosin and/or image-rehearsal therapy with or without cognitive behavioral therapy (CBT) for insomnia [102]. CBT, for those with insomnia, has also been shown to decrease total wake time and improve sleep efficiency [103]. 

**Figure 2:**
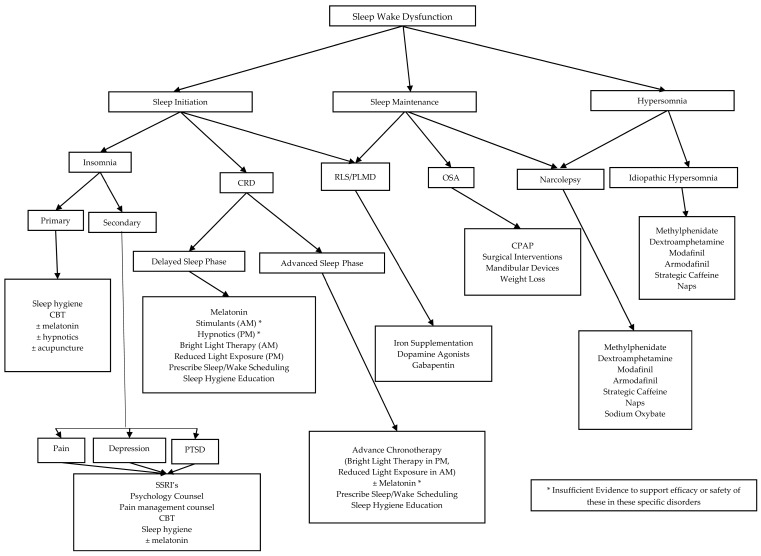
A disease-specific approach to treatment of sleep–wake dysfunction. CRD: Circadian Rhythm Disorder; OSA: Obstructive Sleep Apnea; RLS: Restless Leg Syndrome; PLMD: Periodic Limb Movement Disorder; CBT: Cognitive Behavioral Therapy; SSRI: Selective Serotonin Reuptake Inhibitor

The approach to treating sleep–wake dysfunction is dependent on the specific sleep disorder present (Figure 1 and Figure 2). Melatonin has also been studied for sleep disorders post TBI and although no statistical difference was found with daytime alertness, patients subjectively reported improved daytime alertness compared to baseline [113]. Amitriptyline has also been subjectively reported by patients to help with sleep disorders by increasing their sleep duration, despite any statistical difference being shown [113]. Other adult studies have shown that modafinil and armodafinil significantly improve sleep latency for those with excessive daytime sleepiness (EDS) due to mild or moderate TBI [114,115].

Studies evaluating non-pharmacological treatment are also limited. CBT has been shown to improve children’s behavior post TBI [116]. Similarly, adolescents who participated in an online counselor-assisted problem solving therapy during their post-TBI hospitalization, showed less impaired functioning after [117]. In adults, blue light exposure, as a form of chronotherapy, was shown to reduce fatigue and daytime sleepiness following TBI [118]. Alternative therapies, such as acupuncture, have even demonstrated subjectively improved sleep quality, cognitive function, and the ability to taper sleep medication use [119]. Earlier recommendations include the importance of transition support including alerting school of injury and potential consequences, monitoring students for any increased needs, and offering assistance or adjusting requirements for a couple of weeks post injury [11].

A negative approach to problem solving and depression symptoms has been associated with elevated PTS symptoms and suggests that targeting negative aspects may help mitigate PTS symptoms [120]. This becomes important because adult and childhood survivors of TBI are already at elevated risk of suicidal behavior [121,122,123]. Symptom checklists are not adequate screening tools for all potential psychiatric outcomes, which highlights the importance of the physician’s role in screening for psychiatric disorders and suicidal ideation post injury [26]. 

## 8. Discussion and Future Direction

Traumatic brain injury is a significant pediatric public health concern. It is helpful to view TBI as a disease process, rather than an isolated event [38] due to the cumulative damage that can incur over time. This is evidenced by the features of post-concussive syndrome that can include evolving symptoms of physical, psychological, cognitive, and sleep–wake dysfunction. Increased comorbidity, such as co-occurrence of sleep–wake dysfunction and psychiatric illness, leads to more deleterious outcomes and a more protracted recovery. A multi-disciplinary approach is necessary to provide the comprehensive care necessary in these patients to optimize recovery. 

Perception of an injury and expectations for recovery can dramatically influence patient outcomes [17]. Early incorporation of psychological support should be evaluated as a potential tool for improving outcomes in pediatrics. Adult studies have demonstrated benefit of both pharmacological treatments and non-pharmacological treatments; however, there is still a significant gap in knowledge when it comes to pediatric treatments. A targeted evaluation of these recommendations in patients by age and severity of TBI is necessary to determine whether adult treatments are appropriate and effective. 

Well-defined TBI severity criteria are needed in the pediatric population. In addition, the effect of pre-morbid functioning needs to be better elucidated. Clinical studies that partner with school systems that implement baseline cognitive assessments may help in filling this data void. In order to improve understanding of how sleep and psychiatric symptoms influence recovery, longitudinal studies are needed. These studies should include well defined age at injury to better assess the effects of TBI on normal development and sleep ontogeny. 

## Figures and Tables

**Table 1:** Factors associated with increased risk for youth to experience traumatic brain injuries (TBI) [8,9].

TBI Risk Factors
Low Socioeconomic Status
Overcrowded households
Disadvantaged neighborhoods
High incidence of adverse life events
Young maternal age
Older siblings with few younger siblings
Previous TBI

**Table 2:** Comparison of risk factors for TBI and the development of post-concussive syndrome (PCS) based on retrospective and prospective studies [1,2,3,4,5,6,7].

Retrospective	Prospective	Overlap	Discrepancy
Male gender Lower socioeconomic status (SES) Behavioral problems Attention deficit hyperactivity disorder (ADHD) Cognitive problems Contact Sports Participation Competitive Sports Participation	Male gender Behavioral problems Adverse family events during childhood Punitive parenting practices Maternal depression Maternal age Maternal education	Male Gender Behavioral Problems	SES status Maternal features Cognitive baseline Sports Participation

**Table 3:** Post-concussive symptoms and prevalence [10,11,12,13,14,17,18,19,20,21,22,23].

Post-Concussive Symptoms		Prevalence
**Physical**	Headache	25–47%
	Nausea	7–12%
	Dizziness	30%
	Fatigue	16–40%
	Problems with Balance and Gait	24–34%
	Light and Sound Sensitivity	1–4%
**Emotional**	Emotional Lability	1–40%
	Increased Anxiety	8–17%
**Cognitive**	Cognitive Deficits	7–22%
	Language Impairment	1–68%
	Disorientation and Amnesia	21–30%
**Sleep**	Sleep–Wake Disturbance	13–67%

**Table 4:** TBI comorbidities and associated symptoms [4,32,33,34,35,36,37,38,39,40].

	Diagnoses	Signs and Symptoms
**Sleep–Wake**	Insomnia	Difficulty falling/staying asleep, unrefreshing sleep, insufficient number of hours of sleep despite adequate opportunity
	Sleep Apnea	Snoring, restlessness, apnea, enuresis, diaphoresis, open-mouth breathing, bruxism, sleep fragmentation
	Idiopathic Hypersomnia	Excessive daytime sleepiness, ± excessive number of hours asleep
	Narcolepsy	Excessive daytime sleepiness, cataplexy, sleep paralysis, sleep related hallucinations, sleep fragmentation
	PLMD/RLS *	PLMs >5/h on PSG; Restlessness, discomfort in arms or legs that interferes with sleep onset or maintenance, improves with movement
	CRD	Sleep difficulties that conflict with age typical circadian rhythm; When given opportunity sleeps appropriate number of hours for age
	Parasomnia	Sleep walking, sleep talking, confusional arousals, night terrors, REM behavior disorder/dream enactment behavior
**Psychiatric**	Anxiety	Avoidance, phobias, obsessive compulsive symptoms, generalized anxious feelings
	Depression	Fatigue, irritability, sadness, difficulty concentrating, difficulty with recall, suicidality
	ADHD	Impaired attention, hyperactivity, impaired working memory, impaired working speed
	PTSD	Headaches, decreased psychosocial recovery, sleep disturbance/nightmares, pain, flashbacks, amnesia, irritability/aggression, concentration difficulty

PLMD: periodic limb movement disorder; RLS: restless leg syndrome; CRD: circadian rhythm disorder; ADHD: attention deficit hyperactive disorder; PTSD: post-traumatic stress disorder; PLM: periodic limb movements; PSG: polysomnography; * Note: RLS is a clinical diagnosis and PLMD is a polysomnographic diagnosis.

**Table 5:** Risks factors associated with prolonged recovery following TBI [2,3,41,42,43].

Risk Factors of Protracted Recovery
Pre-injury psychiatry history
Injury Severity
Family dysfunction
Sleep–Wake Dysfunction
Re-injury
Female gender
Referral to Rehabilitation Facility
Prescription for acute headache rescue therapy
Chronic headache treatment
Presenting SCAT2 * score <80
Participation in a non-helmeted sport

* SCAT2—Sport concussion assessment tool.

**Table 6:** Psychiatric disorders and treatments [104,105,106,107,108,109,110].

Psychiatric Disorder	Treatment Options
**Depression**	
Mild	CBT ± Exercise
Severe	CBT + SSRI ± Exercise
Suicidality	CBT + SSRI ± Hospitalization ± Exercise
With psychotic features	CBT + Antidepressant + Antipsychotic ± Exercise
Refractory	CBT + Antidepressant + Antipsychotic ± Exercise ± ECT
**Anxiety**	First Line: CBT ± SSRI, SNRI
	Second Line: CBT + SSRI, ± SNRI
	Third Line: CBT + SSRI + different SSRI or SNRI with Benzodiazepines used as a bridge
	until SSRI becomes effective.
**ADHD**	Stimulants [111,112] (methylphenidate, amphetamine), ± CBT, non-stimulants (atomoxetine, guanfacine, clonidine)
**PTSD**	CBT, Ensure Safety, Treat Comorbidities, ± Antiadrenergic medications (clonidine, guanfacine, or prazosin *)

* Prazosin is preferred in patients with PTSD nightmare disorder. CBT: cognitive behavioral therapy; ECT: electroconvulsive therapy; SSRI: selective serotonin reuptake inhibitor; SNRI selective serotonin norepinephrine reuptake inhibitor.

## References

[1] Eme R. (2012). ADHD: An integration with pediatric traumatic brain injury. Expert Rev. Neurother..

[2] Schachar R.J., Park L.S., Dennis M. (2015). Mental Health Implications of Traumatic Brain Injury (TBI) in Children and Youth. J. Can. Acad. Child Adolesc. Psychiatry.

[3] Veliz P., McCabe S.E., Eckner J.T., Schulenberg J.E. (2017). Prevalence of concussion among us adolescents and correlated factors. JAMA.

[4] Li L., Liu J. (2013). The effect of pediatric traumatic brain injury on behavioral outcomes: A systematic review. Dev. Med. Child Neurol..

[5] Davidson L.L., Hughes S.J., O’Connor P.A. (1988). Preschool behavior problems and subsequent risk of injury. Pediatrics.

[6] Bijur P.E., Haslum M., Golding J. (1990). Cognitive and behavioral sequelae of mild head injury in children. Pediatrics.

[7] McKinlay A., Kyonka E.G., Grace R.C., Horwood L.J., Fergusson D.M., MacFarlane M.R. (2010). An investigation of the pre-injury risk factors associated with children who experience traumatic brain injury. Inj. Prev..

[8] Bijur P., Golding J., Haslum M., Kurzon M. (1988). Behavioral predictors of injury in school-age children. Am. J. Dis. Child..

[9] Max J.E., Schachar R.J., Levin H.S., Ewing-Cobbs L., Chapman S.B., Dennis M., Saunders A., Landis J. (2005). Predictors of attention-deficit/hyperactivity disorder within 6 months after pediatric traumatic brain injury. J. Can. Acad. Child Adolesc. Psychiatry.

[10] Gioia G., Micky C. (2016). Acute Concussion Evaluation (ACE): Physicial/Clincian Office Version. 27 April 2006. https://www.cdc.gov/headsup/pdfs/providers/ace-a.pdf.

[11] Kirkwood M.W., Yeates K.O., Taylor H.G., Randolph C., McCrea M., Anderson V.A. (2008). Management of pediatric mild traumatic brain injury: A neuropsychological review from injury through recovery. Clin. Neuropsychol..

[12] Mittenberg W., Wittner M.S., Miller L.J. (1997). Postconcussion syndrome occurs in children. Neuropsychology.

[13] Ponsford J., Willmott C., Rothwell A., Cameron P., Ayton G., Nelms R., Curran C., Ng K.T. (1999). Cognitive and behavioral outcome following mild traumatic head injury in children. J. Head Trauma Rehabil..

[14] Yeates K.O., Luria J., Bartkowski H., Rusin J., Martin L., Bigler E.D. (1999). Postconcussive symptoms in children with mild closed head injuries. J. Head Trauma Rehabil..

[15] McInnes K., Friesen C.L., MacKenzie D.E., Westwood D.A., Boe S.G. (2017). Mild Traumatic Brain Injury (mTBI) and chronic cognitive impairment: A scoping review. PLoS ONE.

[16] Coronado V.G., Haileyesus T., Cheng T.A., Bell J.M., Haarbauer-Krupa J., Lionbarger M.R., Flores-Herrera J., McGuire L.C., Gilchrist J. (2015). Trends in Sports- and Recreation-Related Traumatic Brain Injuries Treated in US Emergency Departments: The National Electronic Injury Surveillance System-All Injury Program (NEISS-AIP) 2001–2012. J. Head Trauma Rehabil..

[17] Kenzie E.S., Parks E.L., Bigler E.D., Lim M.M., Chesnutt J.C., Wakeland W. (2017). Concussion As a Multi-Scale Complex System: An Interdisciplinary Synthesis of Current Knowledge. Front. Neurol..

[18] Nakase-Richardson R., Sherer M., Barnett S.D., Yablon S.A., Evans C.C., Kretzmer T., Schwartz D.J., Modarres M. (2013). Prospective evaluation of the nature, course, and impact of acute sleep abnormality after traumatic brain injury. Arch. Phys. Med. Rehabil..

[19] Chaput G., Giguere J.F., Chauny J.M., Denis R., Lavigne G. (2009). Relationship among subjective sleep complaints, headaches, and mood alterations following a mild traumatic brain injury. Sleep Med..

[20] Hillier S.L., Sharpe M.H., Metzer J. (1997). Outcomes 5 years post-traumatic brain injury (with further reference to neurophysical impairment and disability). Brain Inj..

[21] Huang C.T., Lin W.C., Ho C.H., Tung L.C., Chu C.C., Chou W., Wang C.H. (2014). Incidence of severe dysphagia after brain surgery in pediatric traumatic brain injury: A nationwide population-based retrospective study. J. Head Trauma Rehabil..

[22] Morgan A., Ward E., Murdoch B., Kennedy B., Murison R. (2003). Incidence, characteristics, and predictive factors for dysphagia after pediatric traumatic brain injury. J. Head Trauma Rehabil..

[23] Max J.E., Keatley E., Wilde E.A., Bigler E.D., Levin H.S., Schachar R.J., Saunders A., Ewing-Cobbs L., Chapman S.B., Dennis M. (2011). Anxiety disorders in children and adolescents in the first six months after traumatic brain injury. J. Neuropsychiatry Clin. Neurosci..

[24] Schwartz L., Taylor H.G., Drotar D., Yeates K.O., Wade S.L., Stancin T. (2003). Long-term behavior problems following pediatric traumatic brain injury: Prevalence, predictors, and correlates. J. Pediatr. Psychol..

[25] Catroppa C., Anderson V.A., Morse S.A., Haritou F., Rosenfeld J.V. (2008). Outcome and predictors of functional recovery 5 years following pediatric traumatic brain injury (TBI). J. Pediatr. Psychol..

[26] Ellis M.J., Ritchie L.J., Koltek M., Hosain S., Cordingley D., Chu S., Selci E., Leiter J., Russell K. (2015). Psychiatric outcomes after pediatric sports-related concussion. J. Neurosurg. Pediatr..

[27] Smyth K., Sandhu S.S., Crawford S., Dewey D., Parboosingh J., Barlow K.M. (2014). The role of serotonin receptor alleles and environmental stressors in the development of post-concussive symptoms after pediatric mild traumatic brain injury. Dev. Med. Child Neurol..

[28] Shay N., Yeates K.O., Walz N.C., Stancin T., Taylor H.G., Beebe D.W., Caldwell C.T., Krivitzky L., Cassedy A., Wade S.L. (2014). Sleep problems and their relationship to cognitive and behavioral outcomes in young children with traumatic brain injury. J. Neurotrauma.

[29] Kostyun R.O., Milewski M.D., Hafeez I. (2015). Sleep disturbance and neurocognitive function during the recovery from a sport-related concussion in adolescents. Am. J. Sports Med..

[30] Hooper S.R., Alexander J., Moore D., Sasser H.C., Laurent S., King J., Bartel S., Callahan B. (2004). Caregiver reports of common symptoms in children following a traumatic brain injury. NeuroRehabilitation.

[31] Keightley M.L., Cote P., Rumney P., Hung R., Carroll L.J., Cancelliere C., Cassidy J.D. (2014). Psychosocial consequences of mild traumatic brain injury in children: Results of a systematic review by the International Collaboration on Mild Traumatic Brain Injury Prognosis. Arch. Phys. Med. Rehabil..

[32] Ornstein T.J., Sagar S., Schachar R.J., Ewing-Cobbs L., Chapman S.B., Dennis M., Saunders A.E., Yang T.T., Levin H.S., Max J.E. (2014). Neuropsychological performance of youth with secondary attention-deficit/hyperactivity disorder 6- and 12-months after traumatic brain injury. J. Int. Neuropsychol. Soc..

[33] Brown E.A., Kenardy J.A., Dow B.L. (2014). PTSD perpetuates pain in children with traumatic brain injury. J. Pediatr. Psychol..

[34] Kovachy B., O’Hara R., Hawkins N., Gershon A., Primeau M.M., Madej J., Carrion V. (2013). Sleep disturbance in pediatric PTSD: Current findings and future directions. J. Clin. Sleep Med..

[35] Ruff R.L., Blake K. (2016). Pathophysiological links between traumatic brain injury and post-traumatic headaches. F1000Research.

[36] Kenardy J., Le Brocque R., Hendrikz J., Iselin G., Anderson V., McKinlay L. (2012). Impact of posttraumatic stress disorder and injury severity on recovery in children with traumatic brain injury. J. Clin. Child Adolesc. Psychol..

[37] Singh K., Morse A.M., Tkachenko N., Kothare S.V. (2016). Sleep Disorders Associated With Traumatic Brain Injury-A Review. Pediatr. Neurol..

[38] Masel B.E., DeWitt D.S. (2010). Traumatic brain injury: A disease process, not an event. J. Neurotrauma.

[39] Bloom D.R., Levin H.S., Ewing-Cobbs L., Saunders A.E., Song J., Fletcher J.M., Kowatch R.A. (2001). Lifetime and novel psychiatric disorders after pediatric traumatic brain injury. J. Can. Acad. Child Adolesc. Psychiatry.

[40] Hajek C.A., Yeates K.O., Gerry Taylor H., Bangert B., Dietrich A., Nuss K.E., Rusin J., Wright M. (2010). Relationships among post-concussive symptoms and symptoms of PTSD in children following mild traumatic brain injury. Brain Inj..

[41] Bock S., Grim R., Barron T.F., Wagenheim A., Hu Y.E., Hendell M., Deitch J., Deibert E. (2015). Factors associated with delayed recovery in athletes with concussion treated at a pediatric neurology concussion clinic. Childs Nerv. Syst..

[42] Miller J.H., Gill C., Kuhn E.N., Rocque B.G., Menendez J.Y., O’Neill J.A., Agee B.S., Brown S.T., Crowther M., Davis R.D. (2016). Predictors of delayed recovery following pediatric sports-related concussion: A case-control study. J. Neurosurg. Pediatr..

[43] Gilbert K.S., Kark S.M., Gehrman P., Bogdanova Y. (2015). Sleep disturbances, TBI and PTSD: Implications for treatment and recovery. Clin. Psychol. Rev..

[44] Zhang J., Xu Z., Zhao K., Chen T., Ye X., Shen Z., Wu Z., Shen X., Li S. (2017). Sleep Habits, Sleep Problems, Sleep Hygiene, and Their Associations With Mental Health Problems Among Adolescents. J. Am. Psychiatr. Nurses Assoc..

[45] Luis C.A., Mittenberg W. (2002). Mood and anxiety disorders following pediatric traumatic brain injury: A prospective study. J. Clin. Exp. Neuropsychol..

[46] Beesdo K., Knappe S., Pine D.S. (2009). Anxiety and anxiety disorders in children and adolescents: Developmental issues and implications for DSM-V. Psychiatr. Clin. N. Am..

[47] Vasa R.A., Gerring J.P., Grados M., Slomine B., Christensen J.R., Rising W., Denckla M.B., Riddle M.A. (2002). Anxiety after severe pediatric closed head injury. J. Can. Acad. Child Adolesc. Psychiatry.

[48] Alfano C.A., Ginsburg G.S., Kingery J.N. (2007). Sleep-related problems among children and adolescents with anxiety disorders. J. Can. Acad. Child Adolesc. Psychiatry.

[49] Weiner C.L., Meredith Elkins R., Pincus D., Comer J. (2015). Anxiety sensitivity and sleep-related problems in anxious youth. J. Anxiety Disord..

[50] Cantor J.B., Bushnik T., Cicerone K., Dijkers M.P., Gordon W., Hammond F.M., Kolakowsky-Hayner S.A., Lequerica A., Nguyen M., Spielman L.A. (2012). Insomnia, fatigue, and sleepiness in the first 2 years after traumatic brain injury: An NIDRR TBI model system module study. J. Head Trauma Rehabil..

[51] Max J.E., Keatley E., Wilde E.A., Bigler E.D., Schachar R.J., Saunders A.E., Ewing-Cobbs L., Chapman S.B., Dennis M., Yang T.T. (2012). Depression in children and adolescents in the first 6 months after traumatic brain injury. Int. J. Dev. Neurosci..

[52] Max J.E., Koele S.L., Smith W.L., Sato Y., Lindgren S.D., Robin D.A., Arndt S. (1998). Psychiatric disorders in children and adolescents after severe traumatic brain injury: A controlled study. J. Can. Acad. Child Adolesc. Psychiatry.

[53] Jorge R.E., Robinson R.G., Moser D., Tateno A., Crespo-Facorro B., Arndt S. (2004). Major depression following traumatic brain injury. Arch. Gen. Psychiatry.

[54] Rao V., McCann U., Han D., Bergey A., Smith M.T. (2014). Does acute TBI-related sleep disturbance predict subsequent neuropsychiatric disturbances?. Brain Inj..

[55] Roberts R.E., Duong H.T. (2014). The prospective association between sleep deprivation and depression among adolescents. Sleep.

[56] Roberts R.E., Duong H.T. (2013). Depression and insomnia among adolescents: A prospective perspective. J. Affect. Disord..

[57] Jorge R.E., Robinson R.G., Arndt S.V., Forrester A.W., Geisler F., Starkstein S.E. (1993). Comparison between acute- and delayed-onset depression following traumatic brain injury. J. Neuropsychiatry Clin. Neurosci..

[58] DiScala C., Lescohier I., Barthel M., Li G. (1998). Injuries to children with attention deficit hyperactivity disorder. Pediatrics.

[59] Hoare P., Beattie T. (2003). Children with attention deficit hyperactivity disorder and attendance at hospital. Eur. J. Emerg. Med..

[60] Bonfield C.M., Lam S., Lin Y., Greene S. (2013). The impact of attention deficit hyperactivity disorder on recovery from mild traumatic brain injury. J. Neurosurg. Pediatr..

[61] Gerring J.P., Brady K.D., Chen A., Vasa R., Grados M., Bandeen-Roche K.J., Bryan R.N., Denckla M.B. (1998). Premorbid prevalence of ADHD and development of secondary ADHD after closed head injury. J. Can. Acad. Child Adolesc. Psychiatry.

[62] Keenan H.T., Hall G.C., Marshall S.W. (2008). Early head injury and attention deficit hyperactivity disorder: Retrospective cohort study. BMJ.

[63] Owens J.A. (2005). The ADHD and sleep conundrum: A review. J. Dev. Behav. Pediatr..

[64] Ekinci O., Okuyaz C., Gunes S., Ekinci N., Orekeci G., Teke H., Cobanogullari Direk M. (2017). Sleep and quality of life in children with traumatic brain injury and ADHD. Int. J. Psychiatry Med..

[65] Bryant R.A., Harvey A.G. (1999). Postconcussive symptoms and posttraumatic stress disorder after mild traumatic brain injury. J. Nerv. Ment. Dis..

[66] Levi R.B., Drotar D., Yeates K.O., Taylor H.G. (1999). Posttraumatic stress symptoms in children following orthopedic or traumatic brain injury. J. Clin. Child Psychol..

[67] de Vries A.P., Kassam-Adams N., Cnaan A., Sherman-Slate E., Gallagher P.R., Winston F.K. (1999). Looking beyond the physical injury: Posttraumatic stress disorder in children and parents after pediatric traffic injury. Pediatrics.

[68] Pinto P.S., Meoded A., Poretti A., Tekes A., Huisman T.A. (2012). The unique features of traumatic brain injury in children. review of the characteristics of the pediatric skull and brain, mechanisms of trauma, patterns of injury, complications, and their imaging findings—Part 2. J. Neuroimaging.

[69] Bryant R. (2011). Post-traumatic stress disorder vs traumatic brain injury. Dialogues Clin. Neurosci..

[70] Guo X., Edmed S.L., Anderson V., Kenardy J. (2017). Neurocognitive predictors of posttraumatic stress disorder symptoms in children 6 months after traumatic brain injury: A prospective study. Neuropsychology.

[71] Ventura T., Harrison-Felix C., Carlson N., Diguiseppi C., Gabella B., Brown A., Devivo M., Whiteneck G. (2010). Mortality after discharge from acute care hospitalization with traumatic brain injury: A population-based study. Arch. Phys. Med. Rehabil..

[72] Macera C.A., Aralis H.J., Rauh M.J., MacGregor A.J. (2013). Do sleep problems mediate the relationship between traumatic brain injury and development of mental health symptoms after deployment?. Sleep.

[73] Viola-Saltzman M., Watson N.F. (2012). Traumatic brain injury and sleep disorders. Neurol. Clin..

[74] Robertson C.L., Scafidi S., McKenna M.C., Fiskum G. (2009). Mitochondrial mechanisms of cell death and neuroprotection in pediatric ischemic and traumatic brain injury. Exp. Neurol..

[75] Kochanek P.M., Clark R.S., Ruppel R.A., Adelson P.D., Bell M.J., Whalen M.J., Robertson C.L., Satchell M.A., Seidberg N.A., Marion D.W. (2000). Biochemical, cellular, and molecular mechanisms in the evolution of secondary damage after severe traumatic brain injury in infants and children: Lessons learned from the bedside. Pediatr. Crit. Care Med..

[76] Dennis M., Guger S., Roncadin C., Barnes M., Schachar R. (2001). Attentional-inhibitory control and social-behavioral regulation after childhood closed head injury: Do biological, developmental, and recovery variables predict outcome?. J. Int. Neuropsychol. Soc..

[77] Sesma H.W., Slomine B.S., Ding R., McCarthy M.L. (2008). Executive functioning in the first year after pediatric traumatic brain injury. Pediatrics.

[78] Tlustos S.J., Peter Chiu C.Y., Walz N.C., Wade S.L. (2015). Neural substrates of inhibitory and emotional processing in adolescents with traumatic brain injury. J. Pediatr. Rehabil. Med..

[79] Saper C.B., Fuller P.M., Pedersen N.P., Lu J., Scammell T.E. (2010). Sleep state switching. Neuron.

[80] Sukumaran T.U. (2011). Pediatric sleep project. Indian Pediatr..

[81] Shekleton J.A., Parcell D.L., Redman J.R., Phipps-Nelson J., Ponsford J.L., Rajaratnam S.M. (2010). Sleep disturbance and melatonin levels following traumatic brain injury. Neurology.

[82] Nardone R., Bergmann J., Kunz A., Caleri F., Seidl M., Tezzon F., Gerstenbrand F., Trinka E., Golaszewski S. (2011). Cortical excitability changes in patients with sleep-wake disturbances after traumatic brain injury. J. Neurotrauma.

[83] Baumann C.R., Stocker R., Imhof H.G., Trentz O., Hersberger M., Mignot E., Bassetti C.L. (2005). Hypocretin-1 (orexin A) deficiency in acute traumatic brain injury. Neurology.

[84] Nishino S., Ripley B., Overeem S., Lammers G.J., Mignot E. (2000). Hypocretin (orexin) deficiency in human narcolepsy. Lancet.

[85] Brundin L., Bjorkqvist M., Petersen A., Traskman-Bendz L. (2007). Reduced orexin levels in the cerebrospinal fluid of suicidal patients with major depressive disorder. Eur. Neuropsychopharmacol..

[86] Schwartz S., Ponz A., Poryazova R., Werth E., Boesiger P., Khatami R., Bassetti C.L. (2008). Abnormal activity in hypothalamus and amygdala during humour processing in human narcolepsy with cataplexy. Brain.

[87] Lin C., Chao H., Li Z., Xu X., Liu Y., Hou L., Liu N., Ji J. (2016). Melatonin attenuates traumatic brain injury-induced inflammation: A possible role for mitophagy. J. Pineal Res..

[88] Reiter R.J., Tan D.X., Kim S.J., Cruz M.H. (2014). Delivery of pineal melatonin to the brain and SCN: Role of canaliculi, cerebrospinal fluid, tanycytes and Virchow-Robin perivascular spaces. Brain Struct. Funct..

[89] Seifman M.A., Adamides A.A., Nguyen P.N., Vallance S.A., Cooper D.J., Kossmann T., Rosenfeld J.V., Morganti-Kossmann M.C. (2008). Endogenous melatonin increases in cerebrospinal fluid of patients after severe traumatic brain injury and correlates with oxidative stress and metabolic disarray. J. Cereb. Blood Flow Metab..

[90] Parcell D.L., Ponsford J.L., Redman J.R., Rajaratnam S.M. (2008). Poor sleep quality and changes in objectively recorded sleep after traumatic brain injury: A preliminary study. Arch. Phys. Med. Rehabil..

[91] Cajochen C., Krauchi K., Mori D., Graw P., Wirz-Justice A. (1997). Melatonin and S-20098 increase REM sleep and wake-up propensity without modifying NREM sleep homeostasis. Am. J. Physiol..

[92] Liberman A.R., Kwon S.B., Vu H.T., Filipowicz A., Ay A., Ingram K.K. (2017). Circadian Clock Model Supports Molecular Link Between PER3 and Human Anxiety. Sci. Rep..

[93] Charrier A., Olliac B., Roubertoux P., Tordjman S. (2017). Clock Genes and Altered Sleep-Wake Rhythms: Their Role in the Development of Psychiatric Disorders. Int. J. Mol. Sci..

[94] Silver J.M., McAllister T.W., Arciniegas D.B. (2009). Depression and cognitive complaints following mild traumatic brain injury. Am. J. Psychiatry.

[95] Alderfer B.S., Arciniegas D.B., Silver J.M. (2005). Treatment of depression following traumatic brain injury. J. Head Trauma Rehabil..

[96] Arciniegas D.B., Anderson C.A., Topkoff J., McAllister T.W. (2005). Mild traumatic brain injury: A neuropsychiatric approach to diagnosis, evaluation, and treatment. Neuropsychiatr. Dis. Treat..

[97] Arciniegas D.B., Silver J.M. (2006). Pharmacotherapy of posttraumatic cognitive impairments. Behav. Neurol..

[98] Levin H., Hanten G., Max J., Li X., Swank P., Ewing-Cobbs L., Dennis M., Menefee D.S., Schachar R. (2007). Symptoms of attention-deficit/hyperactivity disorder following traumatic brain injury in children. J. Dev. Behav. Pediatr..

[99] Willmott C., Ponsford J. (2009). Efficacy of methylphenidate in the rehabilitation of attention following traumatic brain injury: A randomised, crossover, double blind, placebo controlled inpatient trial. J. Neurol. Neurosurg. Psychiatry.

[100] Fann J.R., Leonetti A., Jaffe K., Katon W.J., Cummings P., Thompson R.S. (2002). Psychiatric illness and subsequent traumatic brain injury: A case control study. J. Neurol. Neurosurg. Psychiatry.

[101] Max J.E., Lansing A.E., Koele S.L., Castillo C.S., Bokura H., Schachar R., Collings N., Williams K.E. (2004). Attention deficit hyperactivity disorder in children and adolescents following traumatic brain injury. Dev. Neuropsychol..

[102] Seda G., Sanchez-Ortuno M.M., Welsh C.H., Halbower A.C., Edinger J.D. (2015). Comparative meta-analysis of prazosin and imagery rehearsal therapy for nightmare frequency, sleep quality, and posttraumatic stress. J. Clin. Sleep Med..

[103] Ouellet M.C., Morin C.M. (2007). Efficacy of cognitive-behavioral therapy for insomnia associated with traumatic brain injury: A single-case experimental design. Arch. Phys. Med. Rehabil..

[104] Birmaher B., Brent D., Bernet W., Bukstein O., Walter H., Benson R.S., Chrisman A., Farchione T., Greenhill L., Hamilton J. (2007). Practice parameter for the assessment and treatment of children and adolescents with depressive disorders. J. Can. Acad. Child Adolesc. Psychiatry.

[105] Ginsburg G.S., Becker E.M., Keeton C.P., Sakolsky D., Piacentini J., Albano A.M., Compton S.N., Iyengar S., Sullivan K., Caporino N. (2014). Naturalistic follow-up of youths treated for pediatric anxiety disorders. JAMA Psychiatry.

[106] Ginsburg G.S., Kendall P.C., Sakolsky D., Compton S.N., Piacentini J., Albano A.M., Walkup J.T., Sherrill J., Coffey K.A., Rynn M.A. (2011). Remission after acute treatment in children and adolescents with anxiety disorders: Findings from the CAMS. J. Consult. Clin. Psychol..

[107] Wolraich M., Brown L., Brown R.T., DuPaul G., Earls M., Feldman H.M., Ganiats T.G., Kaplanek B., Meyer B., Perrin J. (2011). ADHD: Clinical practice guideline for the diagnosis, evaluation, and treatment of attention-deficit/hyperactivity disorder in children and adolescents. Pediatrics.

[108] Pliszka S. (2007). Practice parameter for the assessment and treatment of children and adolescents with attention-deficit/hyperactivity disorder. J. Can. Acad. Child Adolesc. Psychiatry.

[109] Pliszka S.R., Crismon M.L., Hughes C.W., Corners C.K., Emslie G.J., Jensen P.S., McCracken J.T., Swanson J.M., Lopez M. (2006). The Texas Children’s Medication Algorithm Project: Revision of the algorithm for pharmacotherapy of attention-deficit/hyperactivity disorder. J. Can. Acad. Child Adolesc. Psychiatry.

[110] Akinsanya A., Marwaha R., Tampi R.R. (2017). Prazosin in Children and Adolescents With Posttraumatic Stress Disorder Who Have Nightmares: A Systematic Review. J. Clin. Psychopharmacol..

[111] Cohen J.A., Mannarino A.P., Murray L.K. (2011). Trauma-focused CBT for youth who experience ongoing traumas. Child Abuse Negl..

[112] Connor D.F., Grasso D.J., Slivinsky M.D., Pearson G.S., Banga A. (2013). An open-label study of guanfacine extended release for traumatic stress related symptoms in children and adolescents. J. Child Adolesc. Psychopharmacol..

[113] Kemp S., Biswas R., Neumann V., Coughlan A. (2004). The value of melatonin for sleep disorders occurring post-head injury: A pilot RCT. Brain Inj..

[114] Menn S.J., Yang R., Lankford A. (2014). Armodafinil for the treatment of excessive sleepiness associated with mild or moderate closed traumatic brain injury: A 12-week, randomized, double-blind study followed by a 12-month open-label extension. J. Clin. Sleep Med..

[115] Kaiser P.R., Valko P.O., Werth E., Thomann J., Meier J., Stocker R., Bassetti C.L., Baumann C.R. (2010). Modafinil ameliorates excessive daytime sleepiness after traumatic brain injury. Neurology.

[116] Pastore V., Colombo K., Liscio M., Galbiati S., Adduci A., Villa F., Strazzer S. (2011). Efficacy of cognitive behavioural therapy for children and adolescents with traumatic brain injury. Disabil. Rehabil..

[117] Wade S.L., Kurowski B.G., Kirkwood M.W., Zhang N., Cassedy A., Brown T.M., Nielsen B., Stancin T., Taylor H.G. (2015). Online problem-solving therapy after traumatic brain injury: A randomized controlled trial. Pediatrics.

[118] Sinclair K.L., Ponsford J.L., Taffe J., Lockley S.W., Rajaratnam S.M. (2014). Randomized controlled trial of light therapy for fatigue following traumatic brain injury. Neurorehabil. Neural Repair.

[119] Zollman F.S., Larson E.B., Wasek-Throm L.K., Cyborski C.M., Bode R.K. (2012). Acupuncture for treatment of insomnia in patients with traumatic brain injury: A pilot intervention study. J. Head Trauma Rehabil..

[120] Rhine T., Cassedy A., Yeates K.O., Taylor H.G., Kirkwood M.W., Wade S.L. (2017). Investigating the Connection Between Traumatic Brain Injury and Posttraumatic Stress Symptoms in Adolescents. J. Head Trauma Rehabil..

[121] Harris E.C., Barraclough B. (1997). Suicide as an outcome for mental disorders. A meta-analysis. Br. J. Psychiatry.

[122] Lewinsohn P.M., Rohde P., Seeley J.R. (1993). Psychosocial characteristics of adolescents with a history of suicide attempt. J. Can. Acad. Child Adolesc. Psychiatry.

[123] Teasdale T.W., Engberg A.W. (2001). Suicide after traumatic brain injury: A population study. J. Neurol. Neurosurg. Psychiatry.

